# Survival Predictors of Heart Rate Variability After Myocardial Infarction With and Without Low Left Ventricular Ejection Fraction

**DOI:** 10.3389/fnins.2021.610955

**Published:** 2021-01-28

**Authors:** Junichiro Hayano, Norihiro Ueda, Masaya Kisohara, Emi Yuda, Robert M. Carney, James A. Blumenthal

**Affiliations:** ^1^Department of Medical Education, Nagoya City University Graduate School of Medical Sciences, Nagoya, Japan; ^2^Tohoku University Graduate School of Engineering, Sendai, Japan; ^3^Department of Psychiatry, Washington University School of Medicine, St. Louis, MO, United States; ^4^Department of Psychiatry, Duke University Medical Center, Durham, NC, United States

**Keywords:** heart rate dynamics, heart rate variability, myocardial Infarction, mortality, redundancy, risk stratification, survival, left ventricular ejection fraction

## Abstract

**Background:**

Heart rate variability (HRV) and heart rate (HR) dynamics are used to predict the survival probability of patients after acute myocardial infarction (AMI), but the association has been established in patients with mixed levels of left ventricular ejection fraction (LVEF).

**Objective:**

We investigated whether the survival predictors of HRV and HR dynamics depend on LVEF after AMI.

**Methods:**

We studied 687 post-AMI patients including 147 with LVEF ≤35% and 540 with LVEF >35%, of which 23 (16%) and 22 (4%) died during the 25 month follow-up period, respectively. None had an implanted cardioverter-defibrillator. From baseline 24 h ECG, the standard deviation (SDNN), root mean square of successive difference (rMSSD), percentage of successive difference >50 ms (pNN50) of normal-to-normal R-R interval, ultra-low (ULF), very-low (VLF), low (LF), and high (HF) frequency power, deceleration capacity (DC), short-term scaling exponent (α_1_), non-Gaussianity index (λ_25__s_), and the amplitude of cyclic variation of HR (Acv) were calculated.

**Results:**

The predictors were categorized into three clusters; DC, SDNN, α_1_, ULF, VLF, LF, and Acv as Cluster 1, λ_25__s_ independently as Cluster 2, and rMSSD, pNN50, and HF as Cluster 3. In univariate analyses, mortality was best predicted by indices belonging to Cluster 1 regardless of LVEF. In multivariate analyses, however, mortality in patients with low LVEF was best predicted by the combinations of Cluster 1 predictors or Cluster 1 and 3 predictors, whereas in patients without low LVEF, it was best predicted by the combinations of Cluster 1 and 2 predictors.

**Conclusion:**

The mortality risk in post-AMI patients with low LVEF is predicted by indices reflecting decreased HRV or HR responsiveness and cardiac parasympathetic dysfunction, whereas in patients without low LVEF, the risk is predicted by a combination of indices that reflect decreased HRV or HR responsiveness and indicator that reflects abrupt large HR changes suggesting sympathetic involvement.

## Introduction

Despite significant achievements in its clinical management (Antman et al., 2004), acute myocardial infarction (AMI) remains a leading cause of death (Virani et al., 2020). AMIs occur in the United States at a rate of 1 person every 40 s, with an associated mortality of approximately 110,000 per year (Virani et al., 2020). Sudden cardiac death (SCD) is the most common cause of death after AMI (Zaman and Kovoor, 2014), and patients with a low left ventricular ejection fraction (LVEF) are at the highest risk of SCD during the early months to years after AMI (Solomon et al., 2005; Adabag et al., 2008). To prevent SCD, prophylactic implantation of cardioverter-defibrillators has been recommended for post-AMI patients with LVEF ≤35% (Moss et al., 2002; Goldberger and Lampert, 2006). However, the generalization of reperfusion therapy early after AMI onset (Aversano et al., 2002) has reduced the proportion of post-AMI patients with low LVEF, and consequently, the majority of SCDs occur in patients with LVEF >35%. It has become more important to find clinical markers to predict an increased risk of death in patients without low LVEF (Arevalo et al., 2016). In this study, we analyzed heart rate variability (HRV) and heart rate (HR) dynamics in post-AMI patients to determine the useful markers and combinations to predict mortality risk separately between patients with LVEF ≤35% and those with LVEF >35%.

The analysis of HRV and HR dynamics are widely used for survival risk stratification in cardiovascular diseases (Camm et al., 1996), particularly after AMI (Kleiger et al., 1987; Bigger et al., 1992; Peng et al., 1995; La Rovere et al., 1998; Huikuri et al., 2000; Bauer et al., 2006; Kantelhardt et al., 2007; Kiyono et al., 2007, 2008; Hayano et al., 2011a, 2017; Watanabe et al., 2016). The R-R interval time series data obtained from the 24 h Holter ECG are mainly used for these analyses and many indices have been proposed. The HRV indices are classified into time-domain and frequency-domain indices (Camm et al., 1996). The time-domain indices include the statistical measures of normal-to-normal (N-N) interval (R-R interval of consecutive sinus rhythms) variation, such as the standard deviation of 24 h N-N interval (SDNN) (Kleiger et al., 1987), root mean square of successive N-N interval difference (rMSSD), percentage of successive N-N intervals differing >50 ms (pNN50), deceleration capacity (DC) (Kantelhardt et al., 2007), and the amplitude of cyclic variation of HR (Acv) (Hayano et al., 2011b). Among these, rMSSD and pNN50 that quantify high-frequency N-N interval fluctuations reflect the tonic or sustained level of cardiac parasympathetic control (Berntson et al., 1997; Laborde et al., 2017). Due to a low-pass filter-like-transfer function, the sympathetic HR control cannot involve the modulation of these high-frequency fluctuations (Berger et al., 1989), and thus, these fluctuations are mediated purely by the vagus. In contrast, Acv reflects the HR responsiveness to apneic episodes during sleep. It quantifies the shortening in cardiac cycles caused by sleep-apnea-induced transient arousals. Because this HR response is abolished by atropine (Zwillich et al., 1982; Guilleminault et al., 1984), Acv is thought to reflect a reflex parasympathetic function. The tonic and reflex parasympathetic dysfunction is believed to be a risk for post-AMI mortality (Camm et al., 1996; Bauer et al., 2006; Hayano et al., 2017) because parasympathetic antagonism against sympathetic activation is important to maintain ventricular myocardial electric stability and to prevent the development of fatal ventricular arrhythmias (Hull et al., 1990, 1994; La Rovere et al., 1998).

The frequency-domain indices of HRV are calculated by the power spectral analysis of N-N interval time series and are quantified as the power of frequency components. Among such components, ultra-low frequency (<0.0033 Hz; ULF) and very-low-frequency (0.0033–0.04 Hz; VLF) components reflect fractal-like HR fluctuation that accounts for most of the power of 24 h HRV (Saul et al., 1988). A reduction in the VLF power is one of the most powerful predictors of post-AMI mortality (Bigger et al., 1992). In contrast, a reduction in the high-frequency component (HF, 0.15–0.40 Hz), which is thought to reflect cardiac parasympathetic dysfunction, paradoxically shows the lowest predictive power (Bigger et al., 1992). This paradox may be explained at least partly by the contamination of non-autonomic high-frequency R-R interval fluctuations caused by heart rate fragmentation (Costa et al., 2017; Hayano et al., 2020), which is a type of pacemaker dysfunction more likely to appear in high-risk patients (Costa et al., 2018).

The HR dynamics reflect the non-linear properties of HR fluctuation. Detrended fluctuation analysis (Peng et al., 1995) quantifies the scaling exponents of fractal-like HR dynamics and a reduction in the short-term (4–11 beats) scaling exponent (α_1_) is increased risk for post-AMI mortality (Huikuri et al., 2000). The non-Gaussianity index (λ) quantifies the probability density function for abrupt large HR changes suggesting sympathetic involvement (Kiyono et al., 2007). The λ is increased in patients with heart failure, known as the state of increased sympathetic activity, while other HRV indices are decreased (Kiyono et al., 2007). Additionally, λ is lower in these patients taking beta-blocker than in those without taking beta-blocker (Kiyono et al., 2007). An increase in λ calculated at a time scale of 25 s (λ_25__s_) predicts increased risk for post-AMI cardiac mortality (Hayano et al., 2011a).

In the present study, we hypothesized that the HRV and HR dynamics indices and their combinations to predict post-AMI mortality risk differ between patients with and without low LVEF (≤35%). Most of earlier studies reporting predictive power of HRV and HR dynamics were conducted in post-AMI patients with mixed levels of LVEF (Kleiger et al., 1987; Bigger et al., 1992; Zuanetti et al., 1996; Lanza et al., 1998; Huikuri et al., 2000; Hayano et al., 2011a). The risk stratification models developed by the earlier studies may need to be reappraised separately depending on LVEF. The prophylactic ICD in post-AMI patients with low LVEF could also modify the risk structures. Considering these factors, we chose 24 h ECG data from the post-AMI cohort collected before ICD became clinically widespread and we compared the HRV and HR dynamics indices associated with mortality risk between patients with and without low LVEF. Furthermore, considering the possible redundancy existing among the indices of HRV and HR dynamics (Yuda et al., 2020), we categorized the indices into classes by cluster analysis and analyzed the class-relationships with mortality risk.

## Materials and Methods

### Study Cohort

We examined retrospective cohort data from a subset of the Enhancing Recovery in Coronary Heart Disease (ENRICHD) study (Berkman et al., 2003) consisting of 687 patients who had an AMI and were admitted to the coronary care units of 4 of the 8 ENRICHD clinical trial sites (Washington University, St. Louis, Missouri; Duke University, Durham, North Carolina; Harvard University, Boston, Massachusetts; Yale University, New Haven, Connecticut) between October 1997 and January 2000. The sample included 327 participants of the ENRICHD clinical trial who scored 10 or higher on the Beck Depression Inventory (Beck, 1972) and 360 AMI control participants who were not randomized in the ENRICHD trial because they were not depressed, but were otherwise medically eligible for the trial. Patients were included if they had analyzable Holter ECG data >20.4 h (85% of 24 h) including >3 h of sleep period (time in bed). Patients were excluded if they: (1) had other life-threatening illnesses; (2) were too ill or logistically unable to participate; (3) had ECG data in sinus rhythm <80% of total recorded beats, or (4) had atrial fibrillation, atrial flutter, or an implanted pacemaker or defibrillator. The collection and analysis of Holter ECG recordings were approved by the ethics committees of the corresponding clinical sites. All participants provided written informed consent to participate in the study.

The end-point of the present study was all-cause mortality. Patients underwent follow-up assessments 6 months after study enrollment and annually thereafter for up to 30 months. The end-points were identified from follow-up visits, telephone calls, routine hospital surveillance, and contacts with patients’ physicians. The records of every identified hospitalization were obtained for review and confirmation by a panel of physicians. Death certificates were obtained for all reported deaths. The mortality endpoints used for the present study were either cardiac deaths (AMI, cardiac failure, and sudden cardiac death) or non-cardiac deaths.

### Measurements

Holter ECGs were recorded for 24 h within 28 [median (IQR), 13 (6–19)] days after the index AMI. The ECG recordings were scanned at the Heart Rate Variability Core laboratory at Washington University on a Marquette SXP Laser scanner with software version 5.8 (Marquette Electronics) using standard procedures. The annotated beat file was exported to a workstation for analysis of HRV and HR dynamics indices.

### Data Analysis

The time-domain and frequency-domain indices of HRV and the non-linear indices of HR dynamics that are known as major predictors of post-AMI mortality were calculated by the methods according to the recommended standard (Camm et al., 1996) and to previously published studies (Peng et al., 1995; Iyengar et al., 1996; Kantelhardt et al., 2007; Kiyono et al., 2007; Hayano et al., 2017).

Briefly, the time series of N-N intervals were derived from 24 h ECG data. For the time domain HRV indices, SDNN was computed as the 24 h standard deviation of N-N intervals, rMSSD was calculated as the square root of the mean square of 24 h successive N-N interval differences, pNN50 was obtained as the percentage of successive N-N intervals differing >50 ms, and DC was computed by the phase rectified signal averaging of the 24 h N-N interval time series (Kantelhardt et al., 2007). Acv was calculated by signal-averaging the amplitude of cyclic variation of HR detected by the method of auto-correlated wave detection with adaptive threshold algorithm (Hayano et al., 2011b).

For the frequency domain index, the N-N interval power spectrum was computed by a Fast Fourier transform with a Hanning window after interpolating with a horizontal step function and resampling at 2 Hz. The power spectral density was integrated for the power within the ULF (<0.0033 Hz), VLF (0.0033–0.04 Hz), LF (0.04–0.15 Hz), and HF (0.15–0.4 Hz) bands, respectively, and transformed into natural logarithmic values.

For the non-linear indices, the fractal correlation properties of HR dynamics were computed using the detrended fluctuation analysis and measured as the short-term (4–11 beat) scaling exponents (α_1_) (Peng et al., 1995; Iyengar et al., 1996). Also, the non-Gaussianity index of λ was calculated at a time scale of 25 s (λ_25__s_) according to our previous work (Hayano et al., 2011a). This analysis detects the intermittency of HR increment. The intermittent behavior of HRV is related to non-Gaussian probability distribution with marked fat tails and a peak around the mean value, indicative of a higher probability of the interspersed appearance of large and small increments than the Gaussian fluctuations. The λ quantifies the fatness of the tails of the non-Gaussian probability distribution. The mathematical description of the non-Gaussianity index has been reported elsewhere (Kiyono et al., 2004, 2007).

### Cluster Analysis of HRV and HR Dynamics Indices

To categorize HRV and HR dynamics indices, a cluster analysis was performed based on the correlation matrix between the indices. We used a divisive type cluster analysis. The analysis started with the assumption that all indices belong to a single cluster and continued to divide clusters until the eigenvalue of the second principal component of all clusters becomes less than 1. The cluster to which the index belongs was determined from the factor structure of the oblique principal component so that the index was classified into the clusters where the first principal component gives the highest correlation coefficient with the index.

### Evaluation of Predictive Performance

The predictive performance of the discriminant models, including those consisting of a single index and those of the combinations of multiple indices, was analyzed by logistic regression and evaluated by Somers’ D and c-statistic. The logistic regression model provided prediction scores for individual participants and compared the scores between all possible pairs of survivors and non-survivors. Pairs with a survivor score higher than non-survivors were considered concordant, otherwise, they were considered discordant. Somers’ D was calculated as the difference between the number of concordant and discordant pairs divided by the number of all possible pairs, taking a value from -1 (all pairs disagree) to 1 (all pairs agree). The c-statistic reflected the area under the receiver-operating characteristic curve for the predictive performance of the regression models.

### Statistical Analysis

We used SAS version 9.4 programs (SAS Institute, Cary, NC). Differences between survivors and non-survivors were evaluated by the Chi-square test for categorical data and by Wilcoxon two-sample test for continuous data. The SAS VARCLUS procedure with an oblique principal component cluster analysis was used to categorize the HRV and HR dynamics indices. The SAS LOGISTIC procedure was used for the logistic regression analysis for mortality risk stratification by HRV and HR dynamics indices and their combinations. All models included age as an independent predictor. For all statistical analyses, *P* < 0.05 was considered significant.

## Results

### Patients’ Characteristics

Patient characteristics are presented in Table 1. With baseline LVEF, the participants were divided into 147 patients with LVEF ≤35% (low LVEF) and 540 patients with LVEF >35%. During the follow-up period, 23 (16%) patients with low LVEF and 22 (4%) patients without low LVEF died from all-causes. Among patients with low LVEF, non-survivors were more often diabetic and mentally depressed, had lower LVEF, and had higher serum creatinine. Survivors were more likely to have had more frequent coronary angioplasty. Among patients without low LVEF, non-survivors were older and more often diabetic and smoker, had more frequent histories of coronary bypass surgery, had lower LVEF, had higher serum creatinine, and were more often Killip class III-IV after the index AMI. Survivors were more likely to have had an index AMI of the inferior wall and had more frequent acute reperfusion after the AMI.

**TABLE 1:** Patients’ characteristics.

	LVEF ≤35%			LVEF >35%		
	Survivor	Non-survivor	P*	Survivor	Non-survivor	P*
Number of patients, *n*	124 (84%)	23 (16%)		518 (96%)	22 (4%)	
**Outcome**						
Follow-up (days), median (IQR)	778 (590–1,024)	499 (175–657)	<0.0001	769 (574–974)	373 (203–696)	<0.0001
Cardiac death	0 (0%)	18 (78%)		0 (0%)	14 (64%)	
**Demographic and clinical**						
Age (years), median (IQR)	62 (53–71)	63 (53–75)	0.3	58 (49–67)	69 (59–71)	0.001
Women	37 (30%)	10 (43%)	0.1	215 (42%)	10 (45%)	0.7
Body mass index (kg/m^2^), median (IQR)	27.6 (24.5–31.2)	28.3 (24.0–35.0)	0.6	28.5 (25.4–32.1)	28.4 (26.2–32.4)	0.6
Hypertension	21 (17%)	4 (17%)	0.9	107 (21%)	8 (36%)	0.05
Diabetes mellitus	32 (26%)	15 (65%)	0.0002	128 (25%)	17 (77%)	<0.0001
Current smoker	38 (31%)	4 (17%)	0.2	189 (36%)	3 (14%)	0.03
BDI score ≥10	52 (42%)	18 (78%)	0.001	243 (47%)	14 (64%)	0.1
History of myocardial infarction	39 (31%)	11 (48%)	0.1	90 (17%)	7 (32%)	0.1
History of coronary bypass surgery	22 (18%)	7 (30%)	0.1	42 (8%)	7 (32%)	<0.0001
LVEF (%), median (IQR)	30 (26–35)	25 (20–30)	0.0007	52 (45–55)	45 (40–52)	0.01
Creatinine (mg/dL), median (IQR)	1.0 (0.8–1.2)	1.3 (1.1–2.3)	<0.0001	1.0(0.8–1.1)	1.2 (0.9–2.1)	0.01
**Index AMI**						
Killip class III-IV	15 (12%)	5 (22%)	0.2	15 (3%)	3 (14%)	0.003
Anterior wall AMI	68 (55%)	9 (39%)	0.2	141 (27%)	4 (18%)	0.4
Inferior wall AMI	42 (34%)	5 (22%)	0.3	258 (50%)	5 (23%)	0.01
**Treatment**						
β-Blockers	101 (81%)	17 (74%)	0.3	424 (82%)	20 (91%)	0.2
Angiotensin converting enzyme inhibitors	90 (73%)	16 (70%)	0.7	222 (43%)	11 (50%)	0.5
Aspirin	110 (89%)	17 (74%)	0.05	476 (92%)	17 (77%)	0.01
Calcium channel blockers	12 (10%)	3 (13%)	0.6	74 (14%)	7 (32%)	0.02
Thrombolytic therapy after AMI	38 (31%)	6 (26%)	0.7	173 (33%)	2 (9%)	0.02
Coronary bypass after AMI	27 (22%)	1 (4%)	0.05	73 (14%)	2 (9%)	0.5
Coronary angioplasty<24 h after AMI	66 (53%)	4 (17%)	0.004	334 (64%)	12 (55%)	0.2
Acute reperfusion < = 12 h after AMI	55 (44%)	6 (26%)	0.1	250 (48%)	5 (23%)	0.02

*Significance of difference by Wilcoxon two sample test for continuous variables and by chi-square test for categorical variables. AMI, acute myocardial infarction; LVEF, left ventricular ejection fraction.

### Cluster Analysis of HRV and HR Dynamics Indices

**FIGURE 1:**
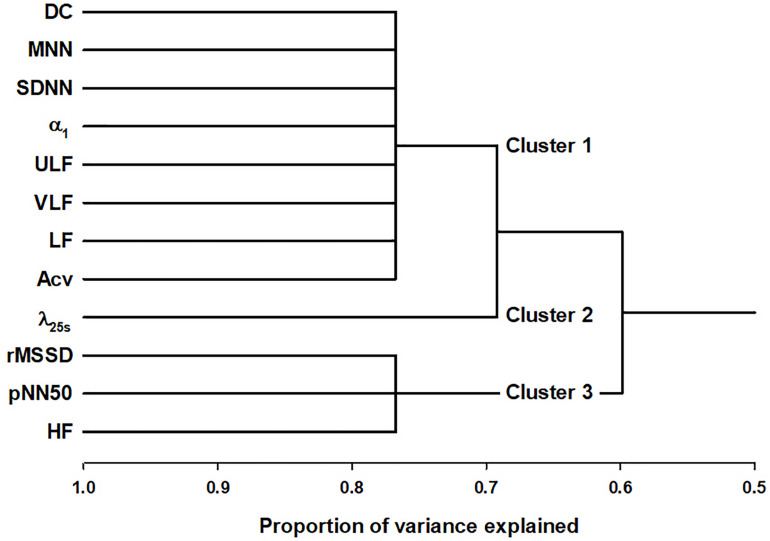
Cluster analysis of HRV and HR dynamics indices in post-AMI patients. DC, deceleration capacity; SDNN, standard deviation of normal-to-normal R-R interval during 24 h; ULF, power of ultra-low-frequency (<0.0033 Hz) component; VLF, power of very-low-frequency (0.0033–0.04 Hz) component; LF, power of low-frequency (0.04–0.15 Hz) component; Acv, amplitude of the cyclic variation of heart rate; λ_25__s_, non-Gaussianity index for a segment length of 25 s; Fcv, frequency of the cyclic variation of heart rate; rMSSD, root mean square of successive R-R interval differences; pNN50, percentage of successive R-R intervals differing >50 ms, and HF, power of high-frequency (0.15–0.40 Hz) component.

Figure 1 shows the tree diagram of the hierarchical cluster based on the principal component of the correlation matrix. The cluster analysis was performed in all 687 patients without separating with LVEF. The predictors were found to be categorized into three clusters; DC, SDNN, α_1_, ULF, VLF, LF, and Acv as Cluster 1, λ_25__s_ independently as Cluster 2, and rMSSD, pNN50, and HF as Cluster 3.

### Univariate Associations of HRV and HR Dynamics With Post-AMI Mortality

Table 2 shows the difference in HRV and HR dynamic indices between survivors and non-survivors. Regardless of LVEF, non-survivors had lower values for all indices in Cluster 1. Among patients with low LVEF, non-survivor has lower HF in Cluster 3, but λ_25__s_ (Cluster 2) did not differ significantly between survivors and non-survivors. Among patients without low LVEF, non-survivors had greater λ_25__s_(Cluster 2), and lower values for all indices in Cluster 3.

**TABLE 2:** Comparisons of baseline heart rate variability (HRV) and heart rate (HR) dynamics indices between survivors and non-survivors.

Index, median (IQR)	LVEF ≤35%			LVEF >35%		
	Survivor	Non-survivor	P*	Survivor	Non-survivor	P*
DC (ms)	4.2(3.1−6.2)	2.9(2.3−3.6)	0.0002	5.3(3.7−6.7)	2.7(2.2−3.8)	<0.0001
SDNN (ms)	87(63−109)	56(47−73)	0.0004	90(69−118)	65(54−78)	0.0008
α_1_	1.2(0.9−1.3)	0.8(0.6−1.1)	0.002	1.2(1−1.3)	0.8(0.7−1.2)	0.001
ULF [ln(ms^2^)]	8.6(7.9−9.1)	7.7(7.3−8.3)	0.0005	8.7(8−9.2)	8(7.6−8.4)	0.001
VLF [ln(ms^2^)]	6.7(6−7.3)	5.7(4.9−6.1)	0.0002	6.8(6−7.5)	5.4(4.6−6.3)	<0.0001
LF[ln(ms^2^)]	5.4(4.3−6.4)	4.1(3.5−4.8)	0.0005	5.6(4.6−6.3)	4.7(2.8−5.3)	0.0003
Acv [ln(ms)]	3.9(3.5−4.3)	3.3(3−3.6)	<0.0001	4.2(3.7−4.5)	3.2(2.9−3.7)	<0.0001
λ_25__s_	0.5(0.5−0.7)	0.6(0.4−0.7)	0.7	0.5(0.5−0.6)	0.6(0.5−0.6)	0.002
rMSSD (ms)	20(14−34)	16(12−24)	0.1	23(16−33)	15(11−29)	0.01
pNN50 (%)	2.6(0.4−10.7)	0.8(0.1−4.8)	0.09	3.1(0.8−10.2)	0.5(0.1−6.8)	0.01
HF [ln(ms^2^)]	4.4(3.5−5.5)	3.6(2.9−4.5)	0.03	4.6(3.9−5.5)	3.5(3−5.2)	0.009

*Significance of difference by Wilcoxon two sample test. Abbreviations are explained in the legend of Figure 1.

Table 3 shows the results of the univariate logistic regression analysis. Regardless of LVEF, the top five predictors based on the c-statistic belonged to Cluster 1.

**TABLE 3:** Predictive power of HRV and HR dynamics indices for post-AMI mortality (logistic regression analysis).

Predictor	LVEF ≤35%				LVEF >35%			
	Concordant,%	Discordant,%	Somers’ D	c-Statistic	Concordant,%	Discordant,%	Somers’ D	c-Statistic
DC	74.4	25.6	0.489	**0.744**	82.7	17.3	0.655	**0.827**
SDNN	75.6	24.4	0.512	**0.756**	77.1	22.9	0.542	0.771
α_1_	70.0	30.0	0.399	0.700	74.7	25.3	0.493	0.747
ULF	74.1	25.9	0.481	0.741	77.7	22.3	0.553	**0.777**
VLF	75.4	24.6	0.508	**0.754**	80.9	19.1	0.618	**0.809**
LF	74.5	25.5	0.490	**0.745**	79.1	20.9	0.582	**0.791**
Acv	80.7	19.3	0.614	**0.807**	82.5	17.5	0.649	**0.825**
λ_25__s_	53.5	46.5	0.070	0.535	74.9	25.1	0.497	0.749
rMSSD	56.5	43.5	0.130	0.565	73.3	26.7	0.465	0.733
pNN50	56.6	43.4	0.133	0.566	73.0	27.0	0.461	0.730
HF	65.4	34.6	0.309	0.654	76.8	23.2	0.536	0.768

**All classification models are adjusted for the effect of age. Boldface indicates the top five largest c-statistic values for LVEF ≥35% and LVEF >35%, respectively. Abbreviations are explained in the legend of Figure 1.**

### Multivariate Associations of HRV and HR Dynamics With Post-AMI Mortality

Table 4 shows the results of logistic regression analyses for all combinations between two predictors. Among patients with low LVEF, the top five performances were observed with the combinations between two predictors both in Cluster 1 and the combination between Cluster 1 and 3 predictors. In contrast, among patients without low LVEF, the top five performances were observed with the combinations between Cluster 1 and 2 predictors.

**TABLE 4:** Predictive performance (c-statistics) of combinations of two predictors among post-AMI patients grouped by LVEF.

	DC	SDNN	α_1_	ULF	VLF	LF	Acv	λ_25__s_	rMSSD	pNN50	HF	
DC	–	0.824	0.823	0.825	0.824	0.823	**0.830**	**0.840**	0.828	0.830	0.824	**LVEF > 35%**
SDNN	0.773	–	0.775	0.778	0.808	0.792	0.824	0.803	0.771	0.772	0.780	
α_1_	0.768	0.773	–	0.782	0.802	0.789	0.816	0.769	0.791	0.792	0.791	
ULF	0.743	0.745	0.726	–	0.810	0.800	0.830	0.815	0.782	0.781	0.792	
VLF	0.758	0.762	0.751	0.754	–	0.809	0.826	**0.832**	0.808	0.808	0.808	
LF	0.765	0.767	0.766	0.742	0.733	–	0.825	**0.832**	0.790	0.791	0.790	
Acv	0.806	**0.810**	**0.811**	0.806	0.807	**0.816**	–	**0.836**	0.824	0.823	0.826	
λ_25__s_	0.728	0.728	0.672	0.739	0.746	0.740	0.807	–	0.801	0.787	0.8327	
rMSSD	0.738	0.769	0.730	0.732	0.761	0.773	**0.816**	0.551	–	0.728	0.769	
pNN50	0.740	0.759	0.737	0.741	0.760	0.765	**0.817**	0.581	0.556	–	0.769	
HF	0.738	0.744	0.768	0.732	0.759	0.751	0.808	0.684	0.717	0.699	–	
**LVEF ≤ 35%**												

**Data are c-statistics calculated by logistic regression analysis. Values in lower left half and upper right half represent those for patients with LVEF ≤35% and those with LVEF > 35%, respectively. Boldface indicates the top five largest values for LVEF ≤35% and LVEF >35%, respectively. All classification models are adjusted for the effect of age. Abbreviations are explained in the legend of Figure 1.**

These features were also observed for the prediction models consisting of three predictors (Table 5). The mortality in patients with low LVEF was best predicted by the combinations of Cluster 1 and 3 predictors. In patients without low LVEF, the top four performances were observed with the combinations between Cluster 1 and 2, although the combinations of Cluster 1, 2, and 3 predictors also showed the 4th best performance.

**TABLE 5:** Combinations of three predictors with the top five predictive performance.

Best combination	Concordant,%	Discordant,%	Somers’ D	c-Statistic
**LVEF ≤35%**				
α_1_ + Acv + rMSSD	82.1	17.9	0.641	0.821
Acv + rMSSD + HF	81.8	18.2	0.636	0.818
Acv + rMSSD + pNN50	81.5	18.5	0.629	0.815
SDNN + Acv + rMSSD	81.5	18.5	0.63	0.815
ULF + Acv + rMSSD	81.5	18.5	0.63	0.815
**LVEF >35%**				
ULF + Acv + λ_25__s_	84.4	15.6	0.689	0.844
VLF + Acv + λ_25__s_	83.7	16.3	0.674	0.837
ULF + VLF + λ_25__s_	83.2	16.8	0.665	0.832
SDNN + VLF + λ_25__s_	83.1	16.9	0.661	0.831
VLF + λ_25__s_ + pNN50	83.1	16.9	0.663	0.831

**All prediction models are adjusted for the effect of age. Abbreviations are explained in the footnote in Table 2.**

## Discussion

In this study, we sought to determine if HRV and HR dynamics indices that predict mortality risk after AMI differ between patients with and without low LVEF (≤35%). Considering the possible redundancy existing among HRV and HR dynamics indices (Yuda et al., 2020), we first categorized the predictors into classes. The cluster analysis revealed that the predictors can be classified into 3 clusters thought to reflect the magnitude of HRV or HR responsiveness (Cluster 1: DC, SDNN, α_1_, ULF, VLF, LF, and Acv), the frequency of abrupt large HR changes (Cluster 2: λ_25__s_), and cardiac parasympathetic function (Cluster 3: rMSSD, pNN50, and HF), respectively. Then, we examined the associations between clustered predictors and mortality risk in patients with and without low LVEF, separately. Univariate analyses showed that mortality was best predicted by indices belonging to Cluster 1 regardless of LVEF, but multivariate analyses showed that mortality in patients with low LVEF was best predicted by the combinations of two Cluster 1 predictors or Cluster 1 and 3 predictors, while in patients without low LVEF, it was best predicted by the combinations of Cluster 1 and 2 predictors. Our findings indicate that the mortality risk in post-AMI patients with low LVEF is predicted by decreased HRV or HR responsiveness and cardiac parasympathetic dysfunction, whereas in patients without low LVEF, the risk is predicted by a combination of decreased HRV or HR responsiveness and increased abrupt large HR changes suggesting sympathetic involvement.

To our knowledge, this is the first study to compare HRV and HR dynamics indices that predict mortality between post-AMI patients with and without low LVEF. Most of earlier studies reporting predictive power of HRV and HR dynamics were conducted in post-AMI patients with mixed levels of LVEF, although they reported the independence of the predictive power of the indices from LVEF (Kleiger et al., 1987; Bigger et al., 1992; Zuanetti et al., 1996; Lanza et al., 1998; Hayano et al., 2011a). Also, Huikuri et al. (2000) examined the predictive value of HRV and HR dynamics in post-AMI patients with LVEF ≤35% and reported that a decrease in α_1_ had greater predictive power of post-AMI mortality than conventional HRV indices. Bauer et al. (2006) demonstrated that a decrease in DC had greater predictive power than SDNN and LVEF and reported that the risk stratification by DC was more useful in patients with LVEF >30% than in those with LVEF ≤30%. Liu et al. (2020) recently reported that decreased SDNN, VLF, and DC were independently associated with increased risk of sudden arrhythmic death in post-AMI patients with LVEF ≤35% and that combination of SDNN, VLF, and DC may help identify a high-risk patient group. Lombardi et al. (1996) compared HRV and HR dynamics indices between post-AMI patients with and without low LVEF and they observed reduced HRV power in the entire frequency range in patients with low LVEF, suggesting diminished responsiveness of sinus node to autonomic modulatory inputs in these patients. None of these studies, however, reported the difference in predictors between post-AMI patients with and without low LVEF.

In this study, we used retrospective cohort data of the ENRICHD study. The patients of this cohort had an AMI and admitted hospital between October 1977 and January 2000. Therefore, the fraction of patients who received a primary percutaneous coronary intervention was low and none of them had an ICD. We chose this cohort to allow comparison of post-AMI patients without low LVEF with a sufficiently sized group of patients with low LVEF whose survival risk is not affected by a prophylactic ICD. Additionally, the sample of this study included a subset of patients enrolled in the ENRICHD trial who had elevated symptoms of depression, which could affect the generalizability of our results. However, the proportion of the depressed patients with BDI scores ≥10 was 47.5%, which is comparable to the reported prevalence of depression (45–47%) in general post-AMI populations (Schleifer et al., 1989; Steeds et al., 2004).

We performed a cluster analysis of HRV and HR dynamics indices in the entire cohort of post-AMI patients. The indices were classified into three clusters and we observed that the associations between the HRV and HR dynamics indices and mortality risk were well explained as class-dependent relationships. These findings provide several insights into the underlying mechanisms.

First, the formation of clusters indicates that there are significant inter-correlations between these indices by the eigenvalue criteria of principal component analysis, supporting our previous finding of a big-data study reporting the substantial redundancy among HRV and HR dynamics indices (Yuda et al., 2020).

Second, the observation that all of the top five univariate predictors of post-AMI mortality belonged to Cluster 1 regardless of LVEF indicates the prognostic significance of the feature(s) common to the indices of this cluster. Although Cluster 1 includes a variety of indices, they commonly reflect the magnitude of HRV, such as SDNN, ULF, VLF, and LF, which are thought to be mediated by interactions between sympathetic and parasympathetic nerve activities, although parasympathetic dysfunction has been thought to be a primary cause of decreased HRV at rest and during sleep (Camm et al., 1996). Earlier studies reported that 92% of VLF power was suppressed by high dose atropine (0.04 mg/kg) (Taylor et al., 1998). DC has been developed to measure the rapid increase in R-R intervals caused only by parasympathetic control (Kantelhardt et al., 2007). The α_1_ increases with atropine and decreases with parasympathetic activation (Tulppo et al., 2001, 2005), although it decreases with increased levels of circulating noradrenaline in healthy men (Tulppo et al., 2001) and increases with β-blocker therapy in patients with heart failure (Lin et al., 2001; Ridha et al., 2002). Acv is thought to reflect a reflex parasympathetic function and its decrease indicates blunted parasympathetic responses to sleep apnea episodes (transient hypoxia, arousal, etc.) (Hayano et al., 2017). Acv is almost completely abolished by 2 mg of intravenous atropine but is unchanged by 5 mg of intravenous propranolol (Guilleminault et al., 1984). These indicate that decreased HRV or HR responsiveness mediated primarily by parasympathetic dysfunction is the most important single feature associated with mortality risk in post-AMI patients with and without low LVEF.

Third, the observation that mortality risk in patients with low LVEF was best predicted by the combinations of indices both in Cluster 1 or those in Cluster 1 and 3 indicates an increased risk of the coexistence of tonic/sustained and reflex parasympathetic disfunction. All of the top five combinations included Acv that reflects a reflex parasympathetic function. The other indices including those in Cluster 3 are thought to reflect the tonic or sustained level of parasympathetic function.

Fourth, the observation that mortality risk in patients without low LVEF was best predicted by the combinations of indices in Cluster 1 and 2 indicates an increased risk of the coexistence of decreased HRV or HR responsiveness and increased abrupt large HR changes. The λ_25__s_ reflects the fatness of tails of the probability density function of the magnitude of abrupt large HR changes. Its increase can occur when the relative frequency of large abrupt HR changes to smaller HR changes increases, suggesting the involvement of transient strong sympathetic activations (Kiyono et al., 2008, 2012). The λ is increased in patients with heart failure and the level of increase is associated with mortality risk, while no other HRV or HR dynamics indices predict it (Kiyono et al., 2007). The λ reflects the relative frequency of large abrupt HR changes but does not depend on the magnitude of HR change itself. Thus, this index could detect relative sympathetic overactivity even under the situation of reduces autonomic responsiveness.

Finally, the different predictive values of Cluster 2 predictor (λ_25__s_) between patients with and without low LVEF may be explained by the presence of overt or subclinical heart failure. In patients with low LVEF, the prognostic value of the indices of sympathetic overactivity could be less because sympathetic nerve activity is increased by heart failure, which most of these patients may have. In patients without low LVEF, the indices of sympathetic overactivity could have greater predictive value because it may reflect the presence or development of heart failure in a part of these patients.

### Limitations

Among Cluster 1 predictors, Acv was the best univariate predictor of post-AMI mortality, but this measure requires a cyclic variation of HR associated with sleep apnea episodes. Nevertheless, Acv was able to be calculated in all post-AMI patients. This is because the Holter ECG data having sleep period (time in bed) <3 h were not included in this study and because Acv can be calculated even in patients with subclinical sleep apnea if at least one episode of cyclic variation of HR is detected during sleep. Assuming cases in which Acv cannot be calculated, we examined logistic regression models excluding Acv, but the results for the relationships between clusters and mortality risk did not change (data not shown). Additionally, although study participants were recruited from four different clinical sites in diverse regions of the US, this study was performed using only one cohort of post-AMI patients. To confirm the present findings, future studies using different cohorts should be performed.

## Conclusion

We investigated whether the survival predictors of HRV and HR dynamics depend on LVEF after AMI. The mortality risk in post-AMI patients with low LVEF is predicted by indices that reflect decreased HRV or HR responsiveness and cardiac parasympathetic dysfunction, whereas in patients without low LVEF, the risk is predicted by a combination of predictors reflecting decreased HRV or HR responsiveness and increased abrupt large HR changes suggesting sympathetic involvement.

## Data Availability Statement

The data analyzed in this study is subject to the following licenses/restrictions: The data from this study are available upon request to the corresponding author. As the data contain potentially identifying or sensitive patient information, the use of the data is limited to the purpose and method of research approved by the ethics committees of the corresponding clinical sites. Requests to access these datasets should be directed to Junichiro Hayano, hayano@acm.org.

## Ethics Statement

The studies involving human participants were reviewed and approved by the research ethics committees of Washington University, St. Louis, Missouri; Duke University, Durham, North Carolina; Harvard University, Boston, Massachusetts; and Yale University, New Haven, Connecticut. The patients/participants provided their written informed consent to participate in this study.

## Author Contributions

JH and EY: conceptualization. JH: methodology, software, and writing—original draft preparation and visualization. EY, NU, and MK: validation. EY: formal analysis. MK and NU: investigation. RC and JB: resources, supervision, and funding acquisition. NU: data curation. EY and JB: writing—review and editing. JH and JB: project administration. All authors have read and agreed to the published version of the manuscript.

## Conflict of Interest

The authors declare that the research was conducted in the absence of any commercial or financial relationships that could be construed as a potential conflict of interest.

## Abbreviations

Acv: amplitude of cyclic variation of heart rate
ALLSTAR: Allostatic State Mapping by Ambulatory ECG Repository
AMI: acute myocardial infarction
DC: deceleration capacity
ENRICHD: Enhancing Recovery in Coronary Heart Disease
Fcv: frequency of cyclic variation of heart rate
HR: heart rate
HRF: heart rate fragmentation
HRV: heart rate variability
SDNN: standard deviation of normal-to-normal R-R interval
VLF: very low frequency.
